# COVID-19 Early Detection in Doctors and Healthcare Workers (CEDiD) study: a cohort study on the feasibility of wearable devices

**DOI:** 10.1136/bmjopen-2024-089598

**Published:** 2025-04-05

**Authors:** Alexander Zargaran, Sara Sousi, Gary Colville, Gill Radcliffe, Rayka Malek, Abdel Douiri, Kariem El-Boghdadly, Gaia Nebbia, Rocio T Martinez Nunez, Anne Greenough

**Affiliations:** 1 King's College London - Guy’s Campus, London, UK; 2 Department of Anaesthesia and Perioperative Medicine, Guy's and St Thomas’ NHS Foundation Trust, London, UK; 3 Department of Infection, Guy’s & St Thomas NHS Trust, London, UK; 4 Department of Infectious Diseases, King's College London - Guy’s Campus, London, UK; 5 Department of Women and Children’s Health, King’s College London, London, UK

**Keywords:** COVID-19, Epidemiology, Randomized Controlled Trial, Wearable Electronic Devices

## Abstract

**Background:**

Infectious agents such as SARS-CoV-2 require strategies to contain outbreaks, particularly in hospitals where the spread of infection is most likely. Biometric monitoring of heart rate, temperature, oxygen saturations and sleep might provide important early warning signs for SARS-CoV-2. This study aimed to determine whether a smart medical device (E4 wristband) and a pulse oximeter used to continuously measure heart rate, skin temperature and oxygen saturation would predict the onset of SARS-CoV-2 infection.

**Methods:**

A single-centre, prospective observational cohort of 30 healthcare workers (HCWs) working in areas at high risk for exposure to SARS-CoV-2 were enrolled. HCWs were tested for SARS-CoV-2 using RT-qPCR of daily self-administered swabs for 30 days. Each participant was asked to wear an E4 wristband to measure changes in their heart rate, skin temperature and sleep throughout the study.

**Results:**

Nine (30%) HCWs (median (range) age of 39 (27–57) years) tested positive for COVID-19. No significant differences were found in the pre-infection and post-infection variations in the heart rate (p=0.31) or skin temperature (p=0.44). Seven of the nine positive subjects reported symptoms at some point during the study period: unusual fatigue (40%), headache (33%) and runny nose (22%) were the most frequent. Analysis of daily trends in observations demonstrated significant fluctuations in biometric parameters.

**Conclusion:**

These results suggest that wearable technology might be useful in documenting signs of SARS-CoV-2 infection in exposed HCWs.

**Trial registration number:**

NCT04363489.

**Strengths and limitations of this study**

- This national clinical trial with ethical approval occurred during a time when the incidence of SARS-CoV-2 infection was high.
- Medical-grade wearable devices were used to monitor data, such as heart rate, skin temperature and sleep.
- Daily laboratory PCR tests were performed to detect infections.
- The sample size (under-recruited) was small.

## Introduction

 Detecting the development of infectious diseases as early as possible is important to ensure timely prevention of spread by initiating early treatment and isolation precautions. COVID-19 pandemic has affected hundreds of millions of people worldwide.^1^ A retrospective analysis of the International Severe Acute Respiratory and Emerging Infections Consortium WHO Clinical Characterisation Protocol United Kingdom study estimated that 11.3% of patients with COVID-19 in 314 UK hospitals became infected after admission. Therefore, hospital-acquired COVID-19 represents an important avenue for research to explore the means of preventing transmission between patients and healthcare workers (HCWs).^2^

Diagnostic assays rely on PCR, lateral flow tests or serological antigen tests. Although these are highly sensitive, there is a lag time in obtaining the results of serology, whilst higher viral loads are required for detection with lateral flow tests.^3^ PCRs require costly reagents, laboratory space, staff and equipment to process samples, whereas lateral flow tests are operator-dependent. These tests are only indicated in the case of significant exposure or clinically significant physiological disturbances, which may be several days into the infection. In addition, asymptomatic cases would go undetected, which a meta-analysis estimated to be 40.5% of all confirmed COVID-19 cases globally.^4^

Wearable healthcare monitoring biometric devices are a rapidly expanding field both in the provision of consumer wearable devices and within health research including COVID-19 and other infectious diseases.^5^,^9^ Devices such as smart watches provide a convenient and accurate means of continuous monitoring of physiological data, which might be sensitive to physiological changes arising from infections.^10 11^ They can provide accurate and continuous measurements of heart rate (HR), skin temperature (TEMP), oxygen saturation, respiratory rate, step count and sleep. Big data in wearable health enables the comparison of deviations in observations with the individual’s baseline, which could be an invaluable tool for supporting clinical decisions.^10^

To date, wearable health research in COVID-19 has retrospectively focused on study participants in a community setting. They have also used consumer devices including the Apple Watch, Fitbit or Garmin.^12 13^ This study aimed to determine physiological changes that could herald the onset of SARS-CoV-2 infection before laboratory confirmation. In addition, the trend in clinical observations and daily self-reported symptoms of COVID-19 were investigated to determine whether an algorithm for early detection of COVID-19 in HCWs could be developed.

## Materials and methods

### Study design

This single-centre, prospective, observational cohort study had two phases: a pilot phase with three participants and a main phase with 27 participants. Data collection ended in June 2021.

### Ethical approval

The study received Health Research Authority and Integrated Research Application System (IRAS) approval on 16/07/2020 (IRAS Project ID 283321; REC Reference: 20/NW/0314). All study participants were assigned a unique trial number, and their data were anonymised, with only the study principal investigator (PI) able to identify participants using the study code. Participants were notified of positive infection results by the study PI. See online supplemental file 1 for the study protocol on data handling.

### Setting

The COVID-19 Early Detection in Doctors and Healthcare Workers (CEDiD) study was conducted at Guy’s and St Thomas’ NHS Foundation Trust, a single-centre setting in the United Kingdom.

### Participants

HCWs who worked in high-risk areas for COVID-19 exposure at Guy’s and St Thomas’ NHS Foundation Trust were included over 30 days.

NHS Health Research Authority and Health and Care Research Wales approvals were obtained (IRAS Project ID 283321; REC Reference 20/NW/0314), and the study was registered at ClinicalTrials.gov (NCT04363489). All participants provided written consent before enrolment.

### Variables

Variables included demographic characteristics, clinical observations, COVID-19 test results and wearable smartwatch data.

### Data sources/measurement

**Figure 1:**
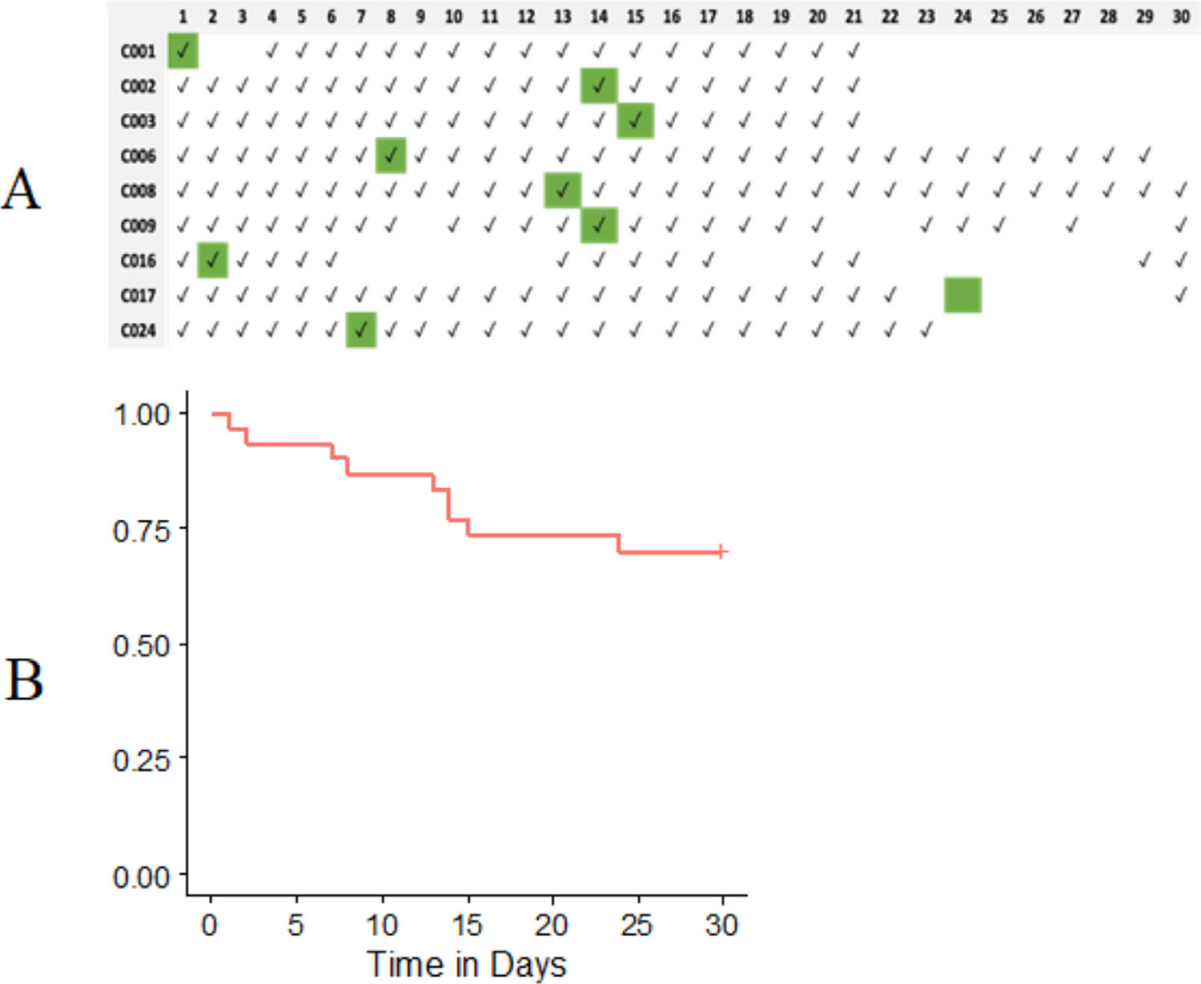
(A) Details on wearing the watch in infected individuals. A tick (✓) represents the days that the individual was wearing their watch, and the green boxes represent the day of a positive test result. (B) Kaplan-Meier survival curve for the time to first positive SARS-CoV-2 infection.

The recruited HCWs were provided with a wearable CE-marked smart medical device (E4 wristband) from Empatica (Empatica, S.R.L. Via Stendhal 36, 20 144 Milan, Italy) worn on their wrists every day during non-working hours for 30 days. The wristband measured the participant’s TEMP, HR and sleep status through electrodermal activity and acceleration. Participants were also given a pulse oximeter to measure their daily oxygen saturations and PCR swabs for self-administered COVID-19 testing every day for 30 days (figure 1). The combined nose and throat swabs for PCR testing of SARS-CoV-2 were obtained following video training. Samples were labelled with the participant’s unique study number, appropriately bagged and dropped off in ward specimen pots. No identifiable information, such as name, surname or date of birth, was included on the label. When participants were not working on a day, they were asked to keep the samples stored in appropriate bags in a cool and dry place until they could deliver them during their next visit to the hospital. Samples were then securely transferred to the laboratory via courier and according to UK government guidelines.

In a participant who tested positive for SARS-CoV-2, they were notified by telephone and advised to self-isolate and contact the hospital’s occupational health department. Participants were followed up further to monitor their symptoms and self-collected swabs until the end of the trial.

The study had two phases: the pilot phase and the main phase. The pilot phase included three participants and assessed the feasibility of the study. After this pilot phase, the protocol was updated to include a further confirmatory PCR test if a single positive PCR test was recorded. The main phase included 27 participants. Data collection ended in June 2021.

### Bias

Efforts were made to minimise bias by providing clear instructions for data collection and testing procedures. Samples were labelled with unique study numbers to maintain anonymity.

### Study size

The sample size calculation was based on established simulations for cohort studies requiring analysis of binary and continuous variable correlation and was set at 60 participants to achieve sufficient power, considering the binary variable of positive vs negative tests versus the continuous variables collected through smartwatch data as well as mitigating for practical considerations such as availability of smartwatches and turnover of staff working in ‘at-risk’ areas.^14^ A significance level of 0.05 was chosen to minimise the risk of type I error, and a power level of 0.80 was targeted to ensure that true effects would be detected.

### Quantitative variables

The following parameters were used for the analysis: TEMP, HR and accelerometer (ACC) assessed at 1, 4 and 32 Hz, respectively. These data were down-sampled to 1 Hz for each variable to facilitate comparison by taking the average, where applicable. The most accurate data were obtained from the devices when the participant was still, which was measured through ACC. To reduce noise, data timestamps were filtered to where ACC was in the interval (0.95, 1.05) g in accordance with the standard error of the device. ACC was defined as $\frac{1}{64}\sqrt{x^{2}+y^{2}+z^{2}}$, where a value of 1 indicated that the wearer was standing still.

Hourly averages were calculated and, where missing, were imputed from the daily average. Daily averages were calculated, and 3-hourly, 6-hourly and 12-hourly averages were calculated for the HR, TEMP and the composite variable of HR/ACC.

### Statistical methods

Descriptive statistics were provided for demographic characteristics of patients, including age, sex and ethnicity, as well as clinical observations. The rate of HCWs who developed SARS-CoV-2 infection over the course of the study was reported in addition to the 95% CI. Student’s t-tests were carried out to detect statistical significance from −7 to +7 days after the first positive test, which was chosen to allow for asymptomatic positive participants. Participants were defined as positive for SARS-CoV-2 if they had two consecutive days of positive PCR tests. The trend in continuous clinical observations, including HR, skin TEMP and sleep, as well as daily self-reported symptoms, was reported. In addition, Kaplan-Meier analysis was used to describe the time-to-event. To determine if differences in clinical observations were significant before and after infection, the Wilcoxon signed-rank test was used. The feasibility of the development of an algorithm for the early detection of COVID-19 in HCWs was investigated. Statistical analyses were conducted using RStudio V.4.2.1. We did not have PPI during study design and development due to the constraints of COVID-19 and associated changes to research practices at the time.

## Results

Between August 2020 and June 2021, we recruited 30 participants. The trial was terminated prematurely, having previously been put on hold, secondary to national policy changes in response to the increasing burden of mortality and morbidity arising from COVID-19 during the height of the pandemic. A total of 30 HCWs were recruited and completed the trial period (table 1). Overall, 9 (30%) of the HCWs tested positive for SARS-CoV-2 by PCR. The infection rate was 3 (95% CI 1.5 to 5.5) in 10 person-months.

**Table 1:** Characteristics of the included participants

Variable	Alln=30	Positiven=9 (30%)	Negativen=2 (70%)
Age	35 (23–57)	39 (27–57)	33 (23–45)
Sex			
Female	22 (73%)	8 (89%)	14 (67%)
Ethnicity			
White	12 (40%)	3 (33%)	9 (43%)
Black	5 (17%)	2 (22%)	3 (14%)
Asian	9 (30%)	1 (11%)	8 (38%)
Mixed	1 (3%)	0 (0%)	1 (5%)
Unknown	3 (10%)	3 (33%)	0 (0%)
Pre-existing medical condition			
Yes	2 (7%)	1 (11%)	1 (5%)
No	25 (83%)	7 (78%)	18 (86%)
Unknown	3 (10%)	1 (11%)	2 (9%)

Data are presented as median (range) or n (%).

### Time to SARS-CoV-2 infection

Data of positive tested individuals wearing their watch throughout the 30-day study period is provided in figure 1A. Survival analysis was performed for time to SARS-CoV-2 infection (figure 1B).

### Wearable data on heart rate, skin temperature and sleep

HR, skin TEMP and sleep hours over the study course were reported for all participants. Data before and after infection were analysed for individuals who tested positive (tables2  3). Pre-COVID-19 was defined as the period up to 7 days prior to a positive test and post-COVID-19 as the period up to 7 days after the positive test. No significant differences in variations of either HR (p=0.31) or skin TEMP (p=0.44) were found in infected individuals pre-COVID-19 and post-COVID-19.

**Table 2:** Pre-COVID-19 and post-COVID-19 wearable data summary for heart rate

Study participant	Heart rate (BPM)Pre-COVID-19			Heart rate (BPM)Post-COVID-19		
	Mean	SD	CV*	Mean	SD	CV
C001	–	–	NA†	89.4	32.3	36.2%
C002	79.9	25.0	31.3%	84.6	24.9	29.4%
C003	81.4	19.8	24.3%	76.9	16.6	21.6%
C006	67.4	11.7	17.4%	64.0	10.7	16.7%
C008	74.8	21.1	28.2%	74.3	20.8	28.0%
C009	81.8	28.7	35.1%	79.5	21.9	27.5%
C016	80.0	18.8	23.5%	69.8	17.9	25.6%
C017	64.3	12.8	19.9%	60.1	4.7	7.9%
C024	66.6	16.7	25.1%	64.2	19.5	30.4%

* The coefficient of variation (CV) is defined as SD/mean.

† There is no pre-COVID-19 data for participant C001 due to the positive test on the first day of the study.

BPM, beats per minute.

**Table 3:** Pre-COVID-19 and post-COVID-19 wearable data summary for skin temperature

Study participant	Skin temp (°C)Pre-COVID-19			Skin temp (°C)Post-COVID-19		
	Mean	SD	CV†	Mean	SD	CV†
C001	–	–	NA*	33.1	5.7	17.3%
C002	33.8	3.8	11.3%	33.5	4.5	13.6%
C003	33.9	3.0	8.8%	34.2	2.6	7.7%
C006	34.0	1.9	5.6%	34.8	1.4	4.1%
C008	33.2	3.0	8.9%	33.7	2.6	7.8%
C009	32.9	4.5	13.5%	33.3	3.5	10.4%
C016	33.9	2.6	7.5%	34.7	3.0	8.5%
C017	34.6	2.6	7.4%	35.2	1.4	4.1%
C024	34.0	1.8	5.2%	34.0	2.7	8.1%

* There is no pre-COVID-19 data for participant C001 due to the positive test on the first day of the study.

† The coefficient of variation (CV) is defined as SD/mean.

Significant differences were noted in the daily comparison of daily TEMP (online supplemental figure 1), HR (online supplemental figure 2) and HR/ACC (online supplemental figure 3) using participants as self-controls. However, no consistent direction of relationships was identified from the nine positive participants. The fact that the devices were worn during non-working hours and while sleeping during the study should mitigate potential confounders of stressors.

### Self-reported health and oxygen saturation

Subjective self-reported health assessed on a 0–10 scale, where 10 denoted feeling completely well and 0 denoted feeling poorly, and oxygen saturation (%SpO2) trends during the study period were investigated (online supplemental figures 4 and 5). While both self-reported health and oxygen saturations demonstrated fluctuations in subjects developing COVID-19, these were not statistically significant, and self-reported health fluctuations were also noted in the non-COVID-19-positive population.

### Symptoms

**Figure 2:**
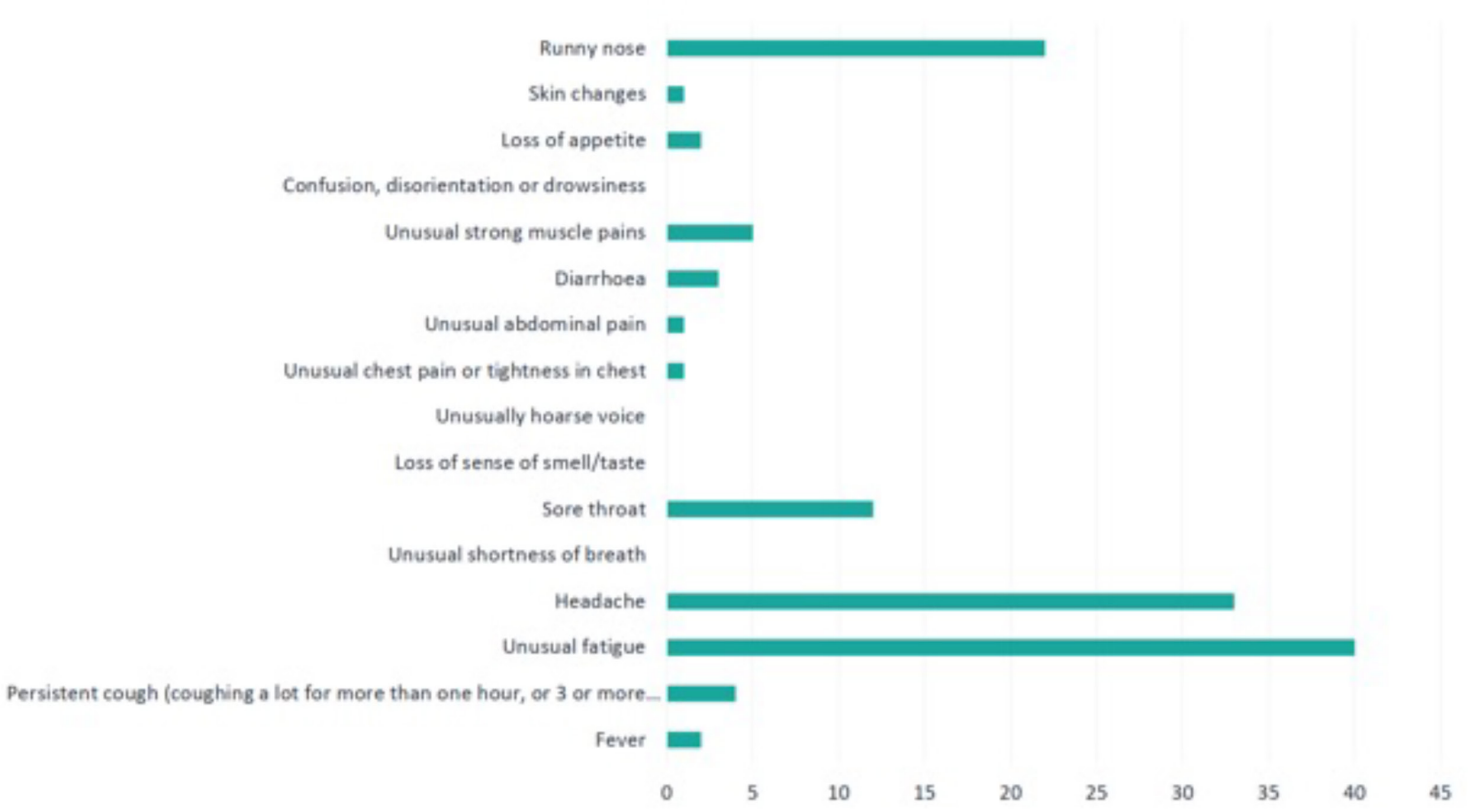
Frequency of self-reported symptoms.

Daily self-reported symptoms were monitored. Of the nine patients diagnosed with COVID-19 during follow-up, seven (78%) reported symptoms during the study period. Five participants (55%) reported symptoms prior to the positive SARS-CoV-2 swab PCR test (between 1 and 4 days). Unusual fatigue (40%), headache (33%) and runny nose (22%) were the most frequent symptoms reported (figure 2).

## Discussion

We report the results of a cohort study assessing the early detection of COVID-19 in HCWs using a combination of self-reported symptoms, PCR tests and medical-grade wearable technology. Significant fluctuations were noted in the observations of study-positive participants; however, no consistent direction or trend was identified.

In this study, a total of 30% of the study population tested positive for SARS-CoV-2, which is higher than the rates of other contemporary wearable health COVID-19 studies.^9 15^ This is likely due to the study demographic being a high-risk population of HCWs who were working on COVID-19-positive wards or intensive care units. The overall population of 30 represents an under-recruitment compared with the power calculation of 60 participants. This was unfortunately due to the disruption to research sustained across all fields during the first two waves of the COVID-19 pandemic.^16^ Our time-to-event analysis demonstrates that COVID-19 can spread unpredictably in clinical areas, with individuals having different susceptibilities to becoming infected. Our attempt to generate an early predictive algorithm for COVID-19 from the vital signs of positive participants was limited by the sample size; however, significant differences were noted, including among the 22% of our study participants who were asymptomatic but positive for COVID-19, reaffirming the role for biometric monitoring for COVID-19.^17 18^

The most common symptoms noted in the present study were unusual fatigue (40%), headache (33%) and runny nose (22%), whereas previous literature highlighted cough, anosmia, fever, shortness of breath and myalgia.^19 20^ No significant difference was noted in the ‘feeling score’ among the study participants. This may have been due to the framing of the question, which sought to identify overall feeling rather than focusing specifically on mental health, as COVID-19 has been noted to have a significant effect on mental health.^21 22^

Hasasneh *et al*^23^ applied an unsupervised clustering approach (K-means and DBSCAN) to consumer-grade Fitbit data in 29 volunteers (11 of whom tested positive), achieving a moderate silhouette score of ~0.55 and~88% accuracy when externally validated. Although this highlights the promise of clustering methods for early infection detection, their reliance on participant-reported infection windows differs from the present study’s daily PCR-based design, which provided more robust confirmation of the presence of infection. In addition, their use of consumer Fitbit data and retrospective data as opposed to the present prospective study, which used a medical-grade device, makes their findings less scientifically robust; however, both studies faced similar problems with sample sizes. Our findings also corroborate the results of the systematic review of 12 observational studies of wearables for COVID-19 detection by Mitratza *et al.*^24^ Their review highlights the methodological heterogeneity across device types (wristbands, rings and patches), retrospective nature of studies and the predominant reliance on self-reported test results rather than daily PCR confirmation of the presence or absence of SARS-CoV-2 RNA. Moreover, while most of the included studies focused on increased HR and TEMP as potential biomarkers, findings were often confounded by inconsistent scientific infection data and lack of prospective validation. This study addresses several of these gaps by employing daily PCR tests**,** using a medical-grade wearable, and following participants prospectively, thereby providing a clearer infection timeline and potentially stronger evidence of early physiological signals. This study provides medical-grade biometric data monitoring through wearables; however, the use of wearable devices in providing biometric data remains in its infancy and requires more robust research to provide stronger validation against clinical standards.

This study has strengths and some limitations, which might explain the lack of significant difference between pre-infection and post-infection variations in the HR and skin TEMP. Continuous virtual monitoring of vital signs represents a potentially transformative means of predicting illness.^7^,^925^ Although this technology can redefine the provision of care through virtual monitoring, it remains susceptible to noise interference, which requires a methodical approach to processing.^26 27^ In this study, data were processed in accordance with manufacturer guidance to eliminate movement artefact, only including times of low acceleration (0.95–1.05 g). A limitation of this study was the size of the sample recruited, which fell short of the originally intended 60 participants. However, we were able to report the overall biometric health of HCWs working in high-risk areas for COVID-19. The smartwatches were only worn during non-working hours, so not providing a complete overview of observations, which may fluctuate depending on the time of day. Self-reported symptoms are subject to reporting bias from participants, but this is a problem for many studies. Whilst we included single test positivity as diagnostic, subjects did not have sustained positive tests, and it is possible that our rate of positivity was over-estimated due to test sensitivity. In addition, the study did not account for antipyretic medications or bedrest for participants who became unwell, which could have affected biometric observations after diagnosis. Finally, the sample size was modest and definitive conclusions cannot be drawn.

## Conclusions

This study reports the biometric health of HCWs working in high-risk areas for COVID-19. Significant fluctuations were noted in the observations of the positive participants; however, no consistent direction or trend was identified. The findings provided an insight into the trends and fluctuations in observations of HCWs working in high-risk areas for COVID-19 who tested positive for the infection.

## Supplementary material

10.1136/bmjopen-2024-089598 online supplemental file 1

## Data Availability

Data are available upon reasonable request.
