# Supplementary material for: COVID-19 Early Detection in Doctors and Healthcare Workers (CEDiD) study: a cohort study on the feasibility of wearable devices
Source: BMJ Open. 2025 Apr 5;15(4):e089598. doi: 10.1136/bmjopen-2024-089598 (PMC11973797; doi:10.1136/bmjopen-2024-089598)

Figure S1: TEMP daily T tests

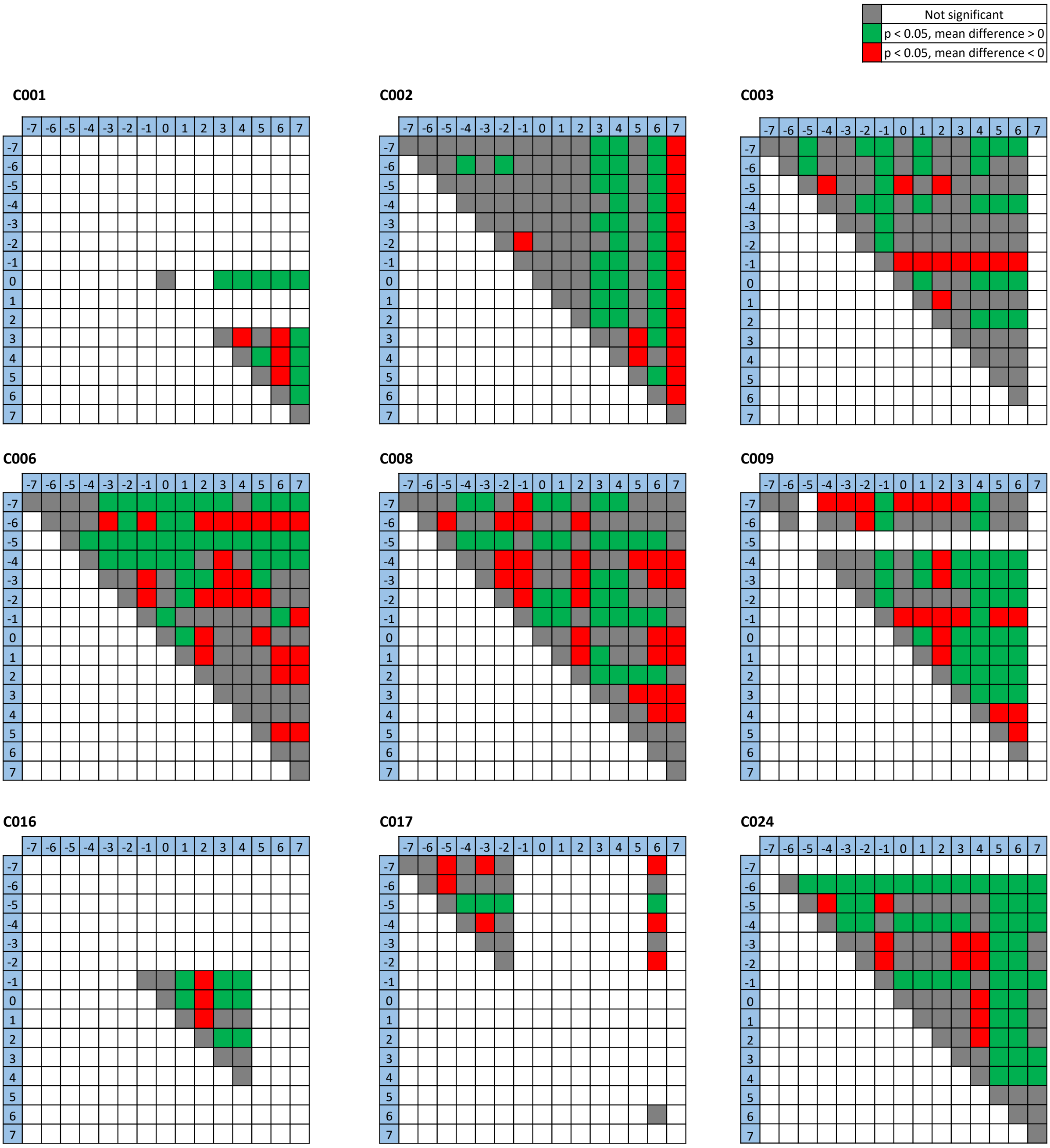

Figure S2: HR daily T tests

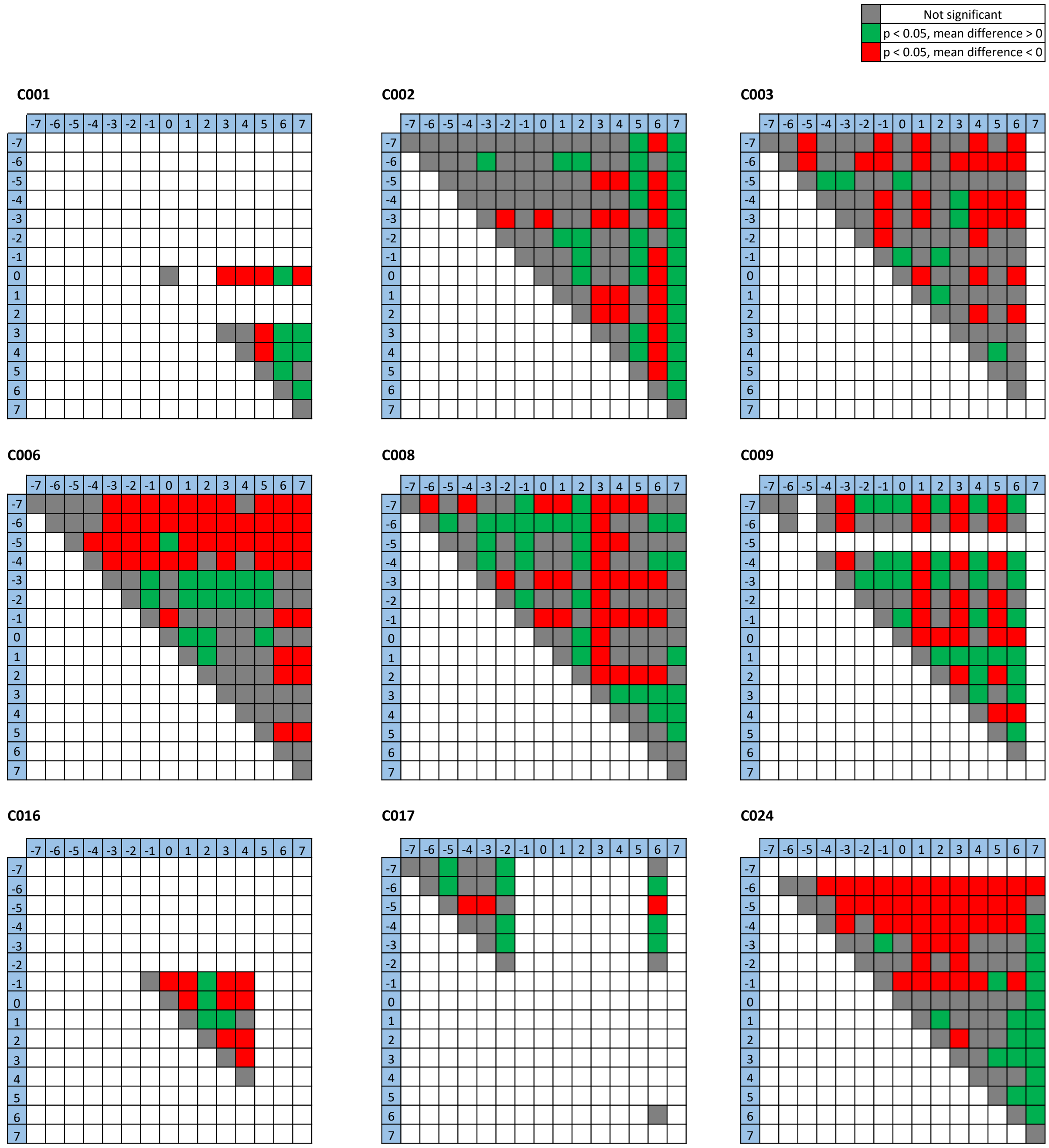

Figure S3: HR/ACC daily T tests

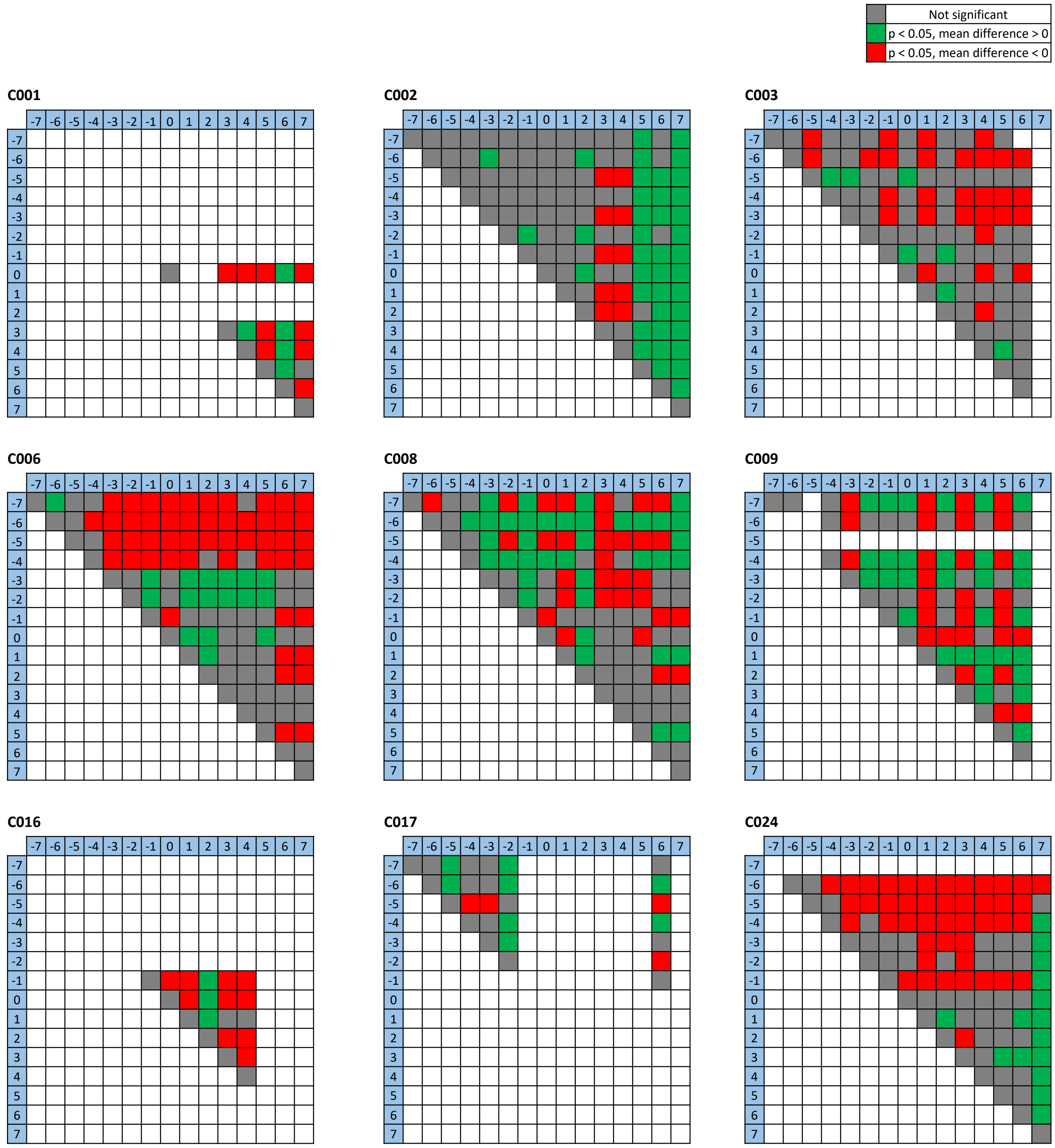

Figures S4: Subjective self-reported health for each individual in the study period

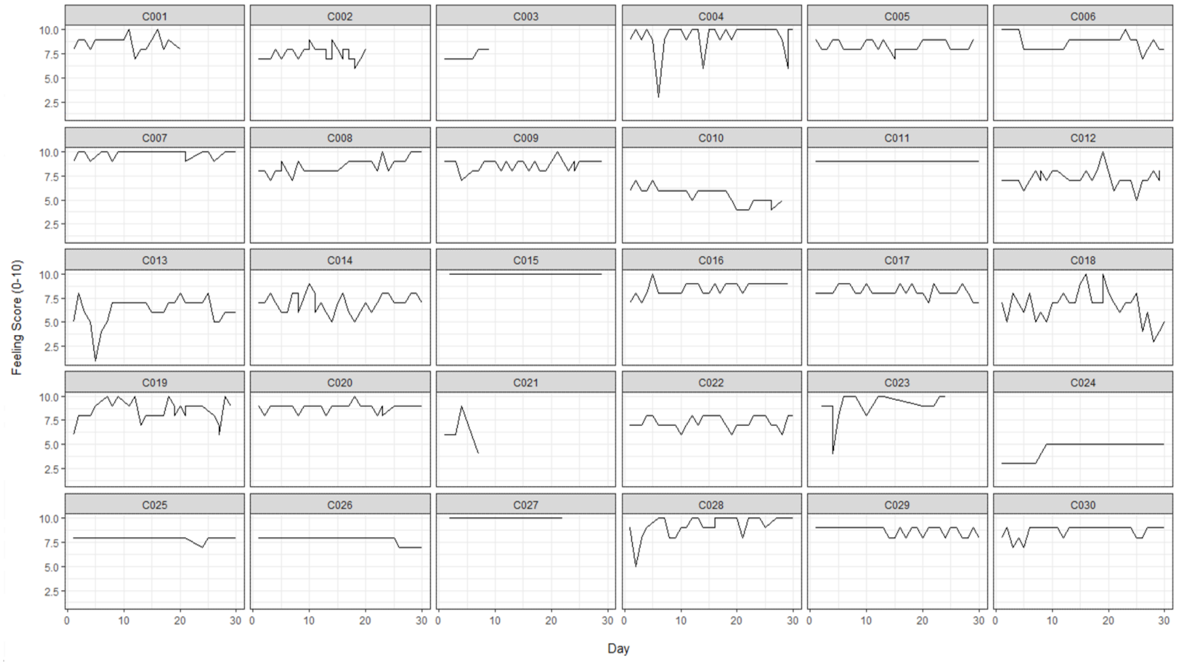

Figure S5: Oxygen saturation for each individual during study period

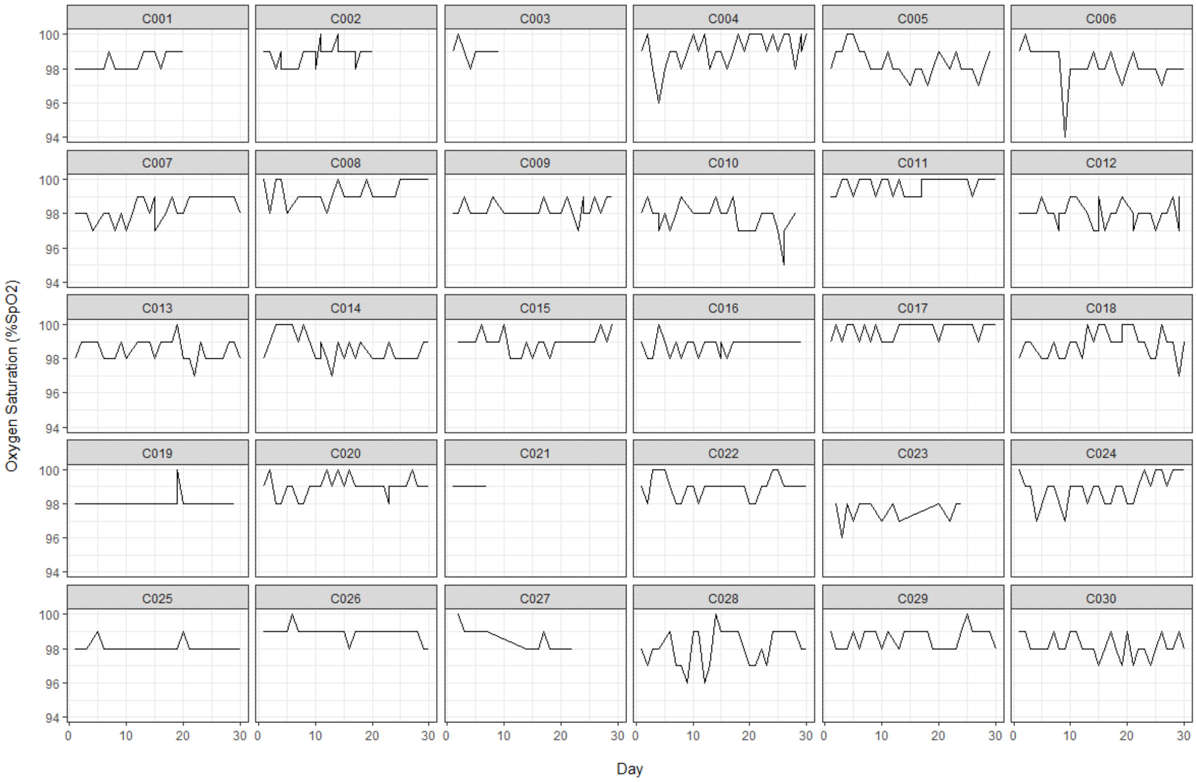

Supplement: online supplemental file 1 [file bmjopen-15-4-s001.pdf]
