# Supplementary material for: COVID-19 Early Detection in Doctors and Healthcare Workers (CEDiD) study: a cohort study on the feasibility of wearable devices
Source: BMJ Open. 2025 Apr 5;15(4):e089598. doi: 10.1136/bmjopen-2024-089598 (PMC11973797; doi:10.1136/bmjopen-2024-089598)
Supplement: Supplementary file 2 [file bmjopen-2024-089598.reviewer_comments.pdf]

PEER REVIEW HISTORY

BMJ Open publishes all reviews undertaken for accepted manuscripts. Reviewers are asked to complete a checklist review form and are provided with free text boxes to elaborate on their assessment. These free text comments are reproduced below.

ARTICLE DETAILS

Title (Provisional)

The COVID-19 early detection in doctors and healthcare workers (CEDiD Study): a cohort study on the feasibility of wearable devices

Authors

Zargaran, Alexander; Sousi, Sara; Colville, Gary; Arbane, Gill; Malek, Rayka; Douiri, Abdel; El Boghdadly, Kariem; Nebbia, Gaia; Martinez Nunez, Rocio; Greenough, Anne

---

VERSION 1 - REVIEW

**Reviewer**	**1**
**Name**	**Miyamori, Daisuke**
**Affiliation**	**Hiroshima Daigaku, Department of General Medicine**
**Date**	**16-Oct-2024**
**COI**	**None**

The manuscript titled "The COVID-19 early detection in doctors and healthcare workers (CEDiD Study): A cohort study" presents an interesting study on the potential of wearable technology for early COVID-19 detection in healthcare workers. However, there are several areas that require improvement to enhance the overall quality and impact of the research. The flow of information, coherence between sections, clarity of key concepts, and logical progression of ideas need attention. Additionally, the manuscript would benefit from a more comprehensive literature review, clearer justification of methodological choices, and a more nuanced discussion of the results in the context of existing research. Addressing these aspects will significantly strengthen the paper's contribution to the field of infectious disease monitoring and early detection.

Flow of Information

Major comments

-The description of wearable technology's role in detecting physiological changes indicative of SARS-CoV-2 infection is somewhat vague in the "Abstract" and "Introduction." Clarify by

specifying the types of physiological changes monitored (e.g., heart rate variability, temperature fluctuations).

-In the abstract and conclusion, authors wrote" Analysis of daily trends in observations demonstrated significant fluctuations in biometric parameters", however no statistical evidence is shown in the manuscript. In addition, no supplemental figures are shown in review manuscripts, thus, we could not assess the figureS1-S5.

-As authors indicated, "This study aimed to determine if there were physiological changes that could herald the onset of SARS-CoV-2 infection before laboratory confirmation", however you conducted analysis between pre- and post-covid infection status after laboratory confirmation. Please clarify the aim of this study.

METHODS

-Sample Size Justification: Expand on how the sample size of 60 was determined to be sufficient for this study's objectives. This could involve a more detailed explanation or simulation results that justify the power calculation.

Result, Self-reported health and Oxygen Saturation

-What symptoms "Subjective self-reported health assessed on a 0-10 scale" assessed? Please explain this.

Discussion

-Interpretation of Non-significant Findings: Discuss the potential reasons for the lack of significant differences in pre- and post-infection variations of heart rate and skin temperature. Speculate on biological or methodological factors that might explain these findings.

-Comparison with Existing Literature: Compare the study's findings with existing research on the effectiveness of wearable technology for early detection of infectious diseases. Highlight any agreements or contradictions and propose explanations for the latter.

Limited clinical data: Lack of detailed clinical outcomes data of COVID-19 infection restricts ability to assess the efficacy of wearable technology. Please consider the treatment administered to HCWs following their COVID-19 diagnosis. If bed rest was prescribed post-diagnosis, it is plausible that the observed increases in pulse rate and other physiological parameters were attenuated, potentially resulting in an underestimation of statistical significance. Additionally, the administration of antipyretic analgesics or other medications may have influenced the estimated difference in results, potentially leading to a reduction in the observed effects.

Minor

Technological Limitations of Wearables: The study mentions processing data to eliminate movement artifact but does not delve into the limitations of wearable technology itself, such as accuracy issues in real-world settings, variability between devices, and how these factors

might influence the data collected. A more thorough discussion on the limitations of the wearable devices used and their validation against clinical standards would add value.

-Limitations: More thoroughly discuss the limitations of the study, including the accuracy of self-reported symptoms.

Lack of vaccination data: The study does not appear to report the vaccination status of participants, making it impossible to assess the impact of vaccination on the study outcomes.

Reviewer **2**

Name **Mukuku, Olivier**

Affiliation **Institut Supérieur des Techniqques Médicales de Lubumbashi, Research**

Date **15-Jan-2025**

COI **None**

This study addresses a timely and highly relevant topic by focusing on the use of wearable technology for the early detection of COVID-19 among healthcare workers, a critical area in pandemic management. It employs an innovative methodology, utilizing wearable devices such as the E4 wristband for continuous biometric monitoring, which presents a scalable and forward-thinking approach. The study is further strengthened by its comprehensive data collection process, combining wearable data with daily self-administered PCR tests to enhance the rigour of the design. Additionally, targeting healthcare workers as the study population ensures its relevance and potential impact on public health policies, given the high-risk nature of this group.

Major Issues:

1. Title and Abstract:

The title could better reflect the limitations and exploratory nature of the study.

Suggestion: Consider a title like, "Feasibility of Wearable Devices for Early Detection of COVID-19 in Healthcare Workers: Insights from a Cohort Study"

2. Sample size and statistical Power: The sample size (30 participants) is significantly below the target (60 participants [page 8]) stated in the methodology. This underpowering limits the reliability of the findings and conclusions drawn.

Suggestion: Acknowledge the limitation in more detail and discuss its implications for the generalizability and statistical significance of the results.

3. Data interpretation: Although fluctuations in biometric parameters were observed, the lack of a consistent pattern or trend weakens the conclusions. The data do not support the interpretation of these fluctuations as early warning signs of COVID-19.

Suggestion: Clarify whether these fluctuations can realistically differentiate between COVID-19 and other potential stressors or illnesses.

4. Inconsistent variables: The study reports significant daily differences in certain parameters (e.g., temperature, and heart rate) but does not identify consistent predictive trends, which reduces the practical utility of the findings.

Suggestion: Consider exploring alternative analytical methods (e.g., machine learning algorithms) that may identify patterns not evident through conventional statistical approaches.

5. Comparison to existing literature: While the study cites other wearable health studies, a more detailed comparison with similar research (e.g., using consumer devices like Fitbit) would contextualize the findings better.

Suggestion: Expand the discussion to include direct comparisons with similar studies to highlight strengths and weaknesses.

6. Lack of external validation: The study lacks validation of its findings in an independent cohort, which raises concerns about reproducibility.

Suggestion: Mention this limitation and propose future research to validate findings in larger and diverse populations.

7. Ethical considerations: The manuscript briefly mentions ethical approvals but does not discuss potential ethical concerns associated with wearable data monitoring, such as privacy and data security.

Suggestion: Include a paragraph presenting these considerations and how they were addressed in the methodology section.

8. Confounding variables: The manuscript does not address potential confounders (e.g., stress, or fatigue unrelated to COVID-19) that could influence biometric data.

Suggestion: Acknowledge and discuss these confounders in the limitations section.

VERSION 1 - AUTHOR RESPONSE

Reviewer: 1
Dr. Daisuke Miyamori, Hiroshima Daigaku
Comments to the Author:
The manuscript titled "The COVID-19 early detection in

doctors and healthcare workers (CEDiD Study): A cohort study" presents an interesting study on the potential of wearable technology for early COVID-19 detection in healthcare workers.

Thank you for your kind comments.

However, there are several areas that require improvement to enhance the overall quality and impact of the research. The flow of information, coherence between sections, clarity of key concepts, and logical progression of ideas need attention. Additionally, the manuscript would benefit from a more comprehensive literature review, clearer justification of methodological choices, and a more nuanced discussion of the results in the context of existing research. Addressing these aspects will significantly strengthen the paper's contribution to the field of infectious disease monitoring and early detection.
Flow of Information

Major comments
-The description of wearable technology's role in detecting physiological changes indicative of SARS-CoV-2 infection is somewhat vague in the "Abstract" and "Introduction." Clarify by specifying the types of physiological changes monitored (e.g., heart rate variability, temperature fluctuations).

We have added a line to the abstract as requested “Each participant was asked to wear an E4 wristband to measure changes in their heart rate, skin temperature and sleep throughout the study”, manuscript lines 46-47

-In the abstract and conclusion, authors wrote” Analysis of daily trends in observations demonstrated significant

fluctuations in biometric parameters”, however no statistical evidence is shown in the manuscript. In addition, no supplemental figures are shown in review manuscripts, thus, we could not assess the figureS1-S5.
We have reattached figures S1-S5 which demonstrate daily fluctuations with data in grey denoting no significant difference, green denoting a significant increase and red denoting a significant reduction. Whilst significant changes across days were identified, no consistent direction of change was observed across the nine positive participants.

-As authors indicated, “This study aimed to determine if there were physiological changes that could herald the onset of SARS-CoV-2 infection before laboratory confirmation”, however you conducted analysis between pre- and post-covid infection status after laboratory confirmation. Please clarify the aim of this study.

To clarify, the laboratory tests were performed daily in order to confirm the presence or absence of infection since at the time of the study laboratory diagnosis was the gold standard for confirmation of infection. This was used to identify participants in the study who developed infection during the study timeframe of 30 days.

METHODS
-Sample Size Justification: Expand on how the sample size of 60 was determined to be sufficient for this study's objectives. This could involve a more detailed explanation or simulation results that justify the power calculation.

The sample size was outlined by the statistician who is a coauthor and included the reference 14 (Teare, M.D.; Dimairo, M.; Shephard, N.; Hayman, A.; Whitehead, A.;

Walters, S.J.;. Sample size requirements to estimate key design parameters from external pilot randomised controlled trials: A simulation study. Trials 2014;15:264), which confirms that for comparable pilot studies a sample size of at least 60 is required to detect a true effect.

Result, Self-reported health and Oxygen Saturation
-What symptoms “Subjective self-reported health assessed on a 0-10 scale” assessed? Please explain this.

The subjective self-reported health assessment from 0-10 was used since at the time of inception of the study, the scientific community did not have a comprehensive understanding of the range of symptoms arising from COVID infection, in order to capture if patients were feeling “off”. We have amended to specify that 10 denoted in perfect health and 0 denoted feeling poorly. In terms of symptoms these are outlined in Figure 2.

Discussion
-Interpretation of Non-significant Findings: Discuss the potential reasons for the lack of significant differences in pre- and post-infection variations of heart rate and skin temperature. Speculate on biological or methodological factors that might explain these findings.
We have accounted for this in our discussion section lines 309-325 and have added a further reason relating to our methodological design of only wearing the watches during non-clinical hours

-Comparison with Existing Literature: Compare the study's findings with existing research on the effectiveness of wearable technology for early detection of infectious diseases. Highlight any agreements or contradictions and

propose explanations for the latter.

We have added discussion on two further studies (REF 26 and 27); a similar sample sized observational study by Hasaneh et al, as well as a systematic review of 12 observational studies by Mitratza et al.

Limited clinical data: Lack of detailed clinical outcomes data of COVID-19 infection restricts ability to assess the efficacy of wearable technology. Please consider the treatment administered to HCWs following their COVID-19 diagnosis. If bed rest was prescribed post-diagnosis, it is plausible that the observed increases in pulse rate and other physiological parameters were attenuated, potentially resulting in an underestimation of statistical significance. Additionally, the administration of antipyretic analgesics or other medications may have influenced the estimated difference in results, potentially leading to a reduction in the observed effects.

We have acknowledged this limitation in our discussion. Unfortunately our study did not place limitations on administration of antipyretic agents or medications or bedrest, and as such this may have had an impact on biometric observations after diagnosis of positivity in the nine participants.

Minor
Technological Limitations of Wearables: The study mentions processing data to eliminate movement artifact but does not delve into the limitations of wearable technology itself, such as accuracy issues in real-world settings, variability between devices, and how these factors might influence the data collected. A more thorough discussion on the limitations of the wearable devices used and their validation against clinical

standards would add value.
We have added a line to clarify that whilst our use of a medical grade device provides robust data from wearables, that biometric wearable data in healthcare remains in its infancy and requires stronger validation against clinical standards.

-Limitations: More thoroughly discuss the limitations of the study, including the accuracy of self-reported symptoms.
We have added more as above and a line to emphasise that self-reported symptoms are subject to reporting bias

Lack of vaccination data: The study does not appear to report the vaccination status of participants, making it impossible to assess the impact of vaccination on the study outcomes.

The data collection phase of the study was performed prior to the introduction of vaccines for COVID-19.

Reviewer: 2
Dr. Olivier Mukuku, Institut Supérieur des Techniqques Médicales de Lubumbashi, Université de Lubumbashi Faculté de Médecine
Comments to the Author:
This study addresses a timely and highly relevant topic by focusing on the use of wearable technology for the early detection of COVID-19 among healthcare workers, a critical area in pandemic management. It employs an innovative methodology, utilizing wearable devices such as the E4 wristband for continuous biometric monitoring, which presents a scalable and forward-thinking approach. The study is further strengthened by its comprehensive data collection process, combining wearable data with daily self-

administered PCR tests to enhance the rigour of the design. Additionally, targeting healthcare workers as the study population ensures its relevance and potential impact on public health policies, given the high-risk nature of this group.

We thank the reviewer for their kind comments

Major Issues:
1. Title and Abstract:
The title could better reflect the limitations and exploratory nature of the study.
Suggestion: Consider a title like, "Feasibility of Wearable Devices for Early Detection of COVID-19 in Healthcare Workers: Insights from a Cohort Study"
We have amended the title to The COVID-19 early detection in doctors and healthcare workers (CEDiD Study): a cohort study on the feasibility of wearable devices. The first part of the title we feel should be included since it is as the study was registered as a national clinical trial

2. Sample size and statistical Power: The sample size (30 participants) is significantly below the target (60 participants [page 8]) stated in the methodology. This underpowering limits the reliability of the findings and conclusions drawn.
Suggestion: Acknowledge the limitation in more detail and discuss its implications for the generalizability and statistical significance of the results.
We have acknowledged this in discussion section lines 317-320.

3. Data interpretation: Although fluctuations in biometric parameters were observed, the lack of a consistent pattern or trend weakens the conclusions. The data do not support the

interpretation of these fluctuations as early warning signs of COVID-19.
Suggestion: Clarify whether these fluctuations can realistically differentiate between COVID-19 and other potential stressors or illnesses.
We have clarified in lines 232-234 that the fact that the devices were worn during non-working hours and whilst sleeping during the course of the study, should mitigate potential confounders of stressors.

4. Inconsistent variables: The study reports significant daily differences in certain parameters (e.g., temperature, and heart rate) but does not identify consistent predictive trends, which reduces the practical utility of the findings.
Suggestion: Consider exploring alternative analytical methods (e.g., machine learning algorithms) that may identify patterns not evident through conventional statistical approaches.

Our study acknowledges the limitations of our findings however we feel it remains an important contribution to the field Because of its setting and design. The machine learning algorithms can observe potential trends, however our study looks at daily fluctuations through recognised statistical methodology in such settings.

5. Comparison to existing literature: While the study cites other wearable health studies, a more detailed comparison with similar research (e.g., using consumer devices like Fitbit) would contextualize the findings better.
Suggestion: Expand the discussion to include direct comparisons with similar studies to highlight strengths and weaknesses.

We have added two further studies (REF 26 and 27) as above
6. Lack of external validation: The study lacks validation of its findings in an independent cohort, which raises concerns about reproducibility.
Suggestion: Mention this limitation and propose future research to validate findings in larger and diverse populations.

The study is a pilot study which requires further larger scale research to validate its findings in larger and more diverse populations

7. Ethical considerations: The manuscript briefly mentions ethical approvals but does not discuss potential ethical concerns associated with wearable data monitoring, such as privacy and data security.
Suggestion: Include a paragraph presenting these considerations and how they were addressed in the methodology section.

We have added a section on ethics in the methods section

8. Confounding variables: The manuscript does not address potential confounders (e.g., stress, or fatigue unrelated to COVID-19) that could influence biometric data.
Suggestion: Acknowledge and discuss these confounders in the limitations section.
We have expanded our discussion to acknowledge further limitations

---

VERSION 2 - REVIEW

**Reviewer**	**2**
**Name**	**Mukuku, Olivier**

Affiliation **Institut Supérieur des Techniqques Médicales de Lubumbashi, Research**

Date **05-Mar-2025**

COI

Please formulate the limitations in sentences in a text and not present them as a list.
